# Enhancing employee wellbeing by ethical leadership in the construction industry: The role of perceived organizational support

**DOI:** 10.3389/fpubh.2022.935557

**Published:** 2022-09-16

**Authors:** Jiajia Cheng, Lianying Zhang, Yuan Lin, Haiyan Guo, Shaoping Zhang

**Affiliations:** ^1^School of Economics and Management, Nanjing Tech University, Nanjing, China; ^2^College of Management and Economics, Tianjin University, Tianjin, China; ^3^MCC Huatian Engineering & Technology Corporation, Nanjing, China; ^4^School of Management, Tianjin University of Commerce, Tianjin, China; ^5^Department of Civil Engineering, Jiangxi Institute of Construction, Nanchang, China

**Keywords:** ethical leadership, employee wellbeing, perceived organizational support, construction industry, mediating role

## Abstract

Employee wellbeing is a crucial determinant in overall organizational performance. However, in the construction Industry, it is damaged by hazardous and stressful work environment. This study aims to explore how ethical leadership influences and thus could enhance employee wellbeing through perceived organizational support (POS). We proposed several hypotheses and developed the research framework accordingly. To test the hypotheses, an elaborately designed survey was used to collect quantitative data from 194 employees in the construction companies in China. Our results show that ethical leadership is positively related to the employee wellbeing. This study further reveals a remarkable indirect effect of ethical leadership on employee wellbeing *via* the mediating POS. Consequently, our findings suggest that, to enhance employee wellbeing, ethical leaders can develop a relaxing ethical environment and provide sufficient organizational support to the employees.

## Introduction

Employee wellbeing describes the employees' senses and perceptions of satisfaction with their work environment and surroundings (1, 2). It is related to psychological and physical health, such as sleep quality and health condition, and greatly influences organizational and individual performance (3–5). However, in the construction industry, employee wellbeing is severely hampered by the hazardous and stressful work environment (6, 7). Employees in such industry are usually stressful with overloaded tasks and extended working hours, tight project deadlines, and inflexible project schedules (8, 9) they would face daily. Additionally, project managers emphasize the outcomes of the projects and tasks more than the endeavors devoted by their employees, even at the expense of their wellbeing. Consequently, their needs are neglected, which will result in the deterioration of their wellbeing (10). These issues attribute to the mindset of seeking maximum benefit or profit at any cost, when profits are valued exceedingly over ethics (11). Therefore, it is urgent to understand the antecedents of employee wellbeing to overcome the challenges in the construction industry.

Previous occupational health studies have argued that ethical leadership is crucial in predicting employee wellbeing and organizational performance in the construction industry (12). Ethical leadership is defined as “the demonstration of normatively appropriate conduct through personal actions and interpersonal relationships, and the promotion of such conduct to followers through two-way communication, reinforcement, and decision-making” (13). Contrary to the mindset of profit at any cost, ethical leadership is valued by adopting explicit ethical aspects to reduce work stress and improve the wellbeing of employees. It advocates that a leader should be a moral person or manager. Specifically, ethical leaders are personally committed to ethical principles (14); or they demonstrate ethical aspects of leadership to affect their employees. Ethical leadership encourages employees to act morally by communication, modeling, punishment, and motivation (9). In this sense, ethical leadership would generate positive outcomes such as the improvement of employee wellbeing (15). Extensive studies have emphasized the positive influence of ethical leadership on employee wellbeing (4, 16). However, ethical leadership is not consistently effective, and its effectiveness can be largely affected by the temporary nature of projects and specific contexts in the construction industry (17). It thereby necessitates substantial organizational support. When leaders' ethical treatments and role modeling are conveyed to employees, it would allow them to work preferably, which would lead to a higher level of wellbeing. Consequently, their perceived organizational support (POS) can effectively cement the relationship between ethical leadership and employee wellbeing.

POS describes that employees develop a general perception involving the degree to which the organization values their contributions and is concerned about their wellbeing (18). It reflects the employees' belief that organizations appreciate their contributions and are highly considerate for their benefits (19). POS demonstrates the positive reciprocity between organizations and employees because employees tend to perform better to repay for the positive effects of their organizations (20). POS is particularly important in the construction industry, because the changing multi-role assignments may lead to unbalanced work-life and a low level of belongingness (21). Project leaders have tried to use ethical leadership to provide control over work or project schedules to motivate the initiatives of employees. However, without considering the particular environment, they fail to efficiently improve employee wellbeing (22). In fact, employees prefer their organizations to recompense for their efforts, value and promote their career development, show great concern for their wellbeing in such a stressful and hazardous work environment (23). Based on the conservation of resources (COR) theory, organizational support is an organizational resource for employees (24). When employees achieve more organizational resources, they will experience more favorable social feelings, such as care, approval, and respect in the workplace (25). POS helps employees develop the sense of belongingness despite their constantly changing work environment. Thus, a significantly efficient way for project managers to enhance employee wellbeing is to provide sufficient organizational support resources. Given the positive effect of POS on employee wellbeing, this study proposes that ethical leadership can effectively enhance employee wellbeing through the mediating mechanism of POS in the construction industry.

Although previous studies have linked ethical leadership to employee wellbeing, little is known about POS mechanisms through which project leaders affect employee wellbeing in the construction industry. Based on the COR theory, this study considers organizational support a valuable organizational resource. Employees receiving the organizational resources are expected to maintain their wellbeing. This study can provide significant implications for project managers to enhance employee wellbeing by accumulating organizational resources.

## Hypotheses development

### Ethical leadership and employee wellbeing

Ethical leaders instill ethical principles (such as trust, consideration, and fairness) into their project management practices to enhance employee wellbeing in the construction industry (26). They make fair and balanced decisions on performance evaluation, genuinely care about employees' needs and develop ethical climate for them, to improve organizational functioning and employee wellbeing (27). Rantika and Yustina (28) considered that when employees perceive ethical leadership, they will show greater work engagement and reduced emotional exhaustion, thus enhanced wellbeing (28). Accordingly, we propose hypothesis (H1).

H1: Ethical leadership has a positive influence on employee wellbeing.

### POS and employee wellbeing

POS helps employees develop positive emotions and feelings when they perceive the organizational approval and support of their abilities, and understanding from their leaders and coworkers (24, 29, 30). According to the principles of reciprocity, employees will perform better in the workplace when they receive more organizational resources. Han et al. argued that employees develop their understanding of organizational goals for work-related efforts and such organizational support improves employees' engagement in the workplace (31). It confirms the critical role of organizational support in developing a favorable environment and opportunities for employees to enhance their wellbeing. Therefore, employees who perceive support from their organizations are more likely to maintain their wellbeing. Thus, hypothesis 2 (H2) is proposed as follows.

H2: POS has a positive influence on employee wellbeing in the construction industry.

### Ethical leadership and POS

Ethical leadership is an antecedent of employees' ethical treatment, which results in high-level POS (32). Credo et al. demonstrated that ethical leaders usually exhibit honesty, trust, fairness, and consideration for their employees (33). Accordingly, employees will view those ethical treatments as experiences of organizational support. Tan et al. argued that ethical leaders can exhibit moral behaviors such as conducting honestly, fairly, and compassionately for POS to emerge (25). Furthermore, moral behaviors and ethical treatments become beneficial organizational resources. When employees understand ethical leadership, levels of POS will be increased. Therefore, we propose hypothesis 3 (H3) as follows.

H3: Ethical leadership has a positive influence on POS in the construction industry.

### The mediating role of POS

Although ethical leadership positively affects employee wellbeing, this effect is facilitated by the mediating POS in the construction industry. Suifan et al. demonstrated that if employees receive sufficient support and recognition from their organizations and leaders, they will develop a strong sense of responsibility to strive to achieve organizations' goals and long-term development (30). Furthermore, POS fosters employees' belief that their organizations value their endeavors, leading to a high level of employee wellbeing. Based on the COR theory, Tan et al. considered that POS is a socioemotional resource such as respect, approval, and caring, which enhances employee wellbeing (25). Hence, hypothesis 4 (H4) is proposed as follows.

H4: POS mediates the relationship between ethical leadership and employee wellbeing in the construction industry.

**Figure 1:**
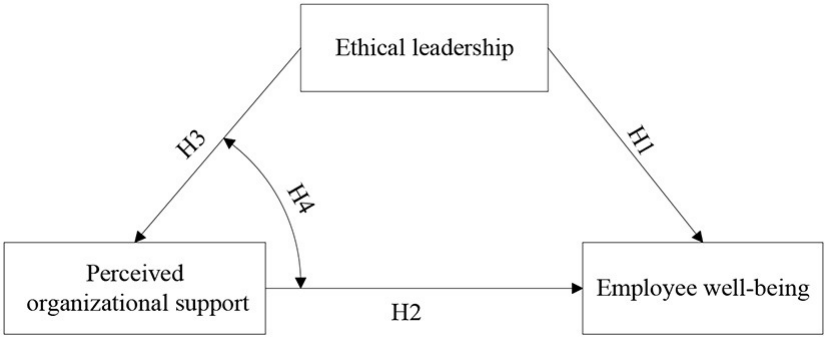
Research framework.

The research framework is presented in Figure 1.

## Research method

### Research design

Following positivist paradigm by Grix, this study adopted quantitative methods to test hypothetical–deductive generalizations (34), which are suitable for gathering extensive knowledge to propose hypotheses. This study collected quantitative data for the measurement items of each variable (i.e., ethical leadership, organizational support, and employee wellbeing) through an elaborately designed questionnaire. The questionnaire mainly included two sections: section one showed the basic information of respondents; section two asked the respondents to evaluate the questionnaire items through a 5-Point Likert-type scale (“1 = strongly disagree” and “5 = strongly agree”). Notably, one item of POS is scored reversely, for example, “if given the opportunity, the organization would take advantage of me.” When the reversed item is strongly agreed, the degree of POS will be relatively low. All items involved important questions presenting ethical leadership, organizational support, and employee wellbeing in this study. All items were obtained and revised from literature about the specific construction industry.

The target samples for this study were full-time employees who came from construction companies in Jiangsu, Henan, Shanxi, Jiangxi provinces and Tianjin, which were selected from the China Construction Industry Association. Most of the construction companies conducted projects regarding housing, subway, and metallurgical plants, which were infamous for the hazard and stressful work environment. Employees from those companies were assigned to at least one project site. In this research, we aimed to study the wellbeing of employees. Thus, the level of analysis of this research was each employee at individual level. Besides, each employee had an immediate manager. Accordingly, they could well describe their feelings about their immediate managers in their daily work.

Data were collected from October 2021 to January 2022. We directly distributed the questionnaires to participants who are acquainted with or have cooperated with the authors. To increase the number of participants, this study adopted “snowballing” method by which available participants were asked for assistance in distributing questionnaires to other respondents. Totally, 241 questionnaires were distributed to employees *via* online social media platforms, such as WeChat, E-mail, or QQ. Due to settings of the online survey, all the returned questionnaires were completed. Finally, 194 usable survey questionnaires were collected, with a response rate of 80.49%. As shown in Appendix 1, 6.7% of the respondents were senior managers, 19.6% were project managers, and other positions occupied were senior specialists (21.6%) in companies and general employees (52.1%). Additionally, of the 194 informants, 34.2% had construction project experience for <5 years, 21.9% for 6 to 10 years, 25% for 11 to 15 years and 18.9% for more than 16 years.

### Measurements

All the variables were measured by a 5-point Likert-type scale, ranging from “totally disagree” = 1 to “totally agree” = 5. The measures of this study are shown in Appendix 3.

Ethical leadership was measured using the 10-item scale built by Brown et al., which has been most widely adopted in the research of project management (13). The sample item involves “My leader sets an example of how to do things the right way in terms of ethics”.

POS was measured using the 4-item scale developed by Cheng et al. (35). Sample items involve “The organization strongly considers my goals and values”, and “The organization shows great concern for me”.

Employee wellbeing was measured by the 9-item scale developed by Sharma et al. (36). The sample items include “How satisfied are you with your health?” and “How satisfied are you with your sleep?”

Control variables including gender and age and work position were set as follows. (1) gender: 1 = male, 2 = female; (2) age: 1 ≤ 25 years, 2 = 26–35 years, 3 = 36–45 years); (3) work position (1 = senior manager, 2 = project manager, 3 = general employee, 4=senior specialist). We controlled these variables since they were shown to influence the employees' perception of wellbeing and organizational support (20, 26) and thereby the relationships among each variable.

### Reliability and validity

This study adopted Cronbach's alpha coefficient to test the internal consistency of each construct. All of the Cronbach's alpha coefficients exceed 0.7 (ranging from 0.850 to 0.937), which demonstrate an acceptable level of reliability. The Cronbach's alpha coefficient tested the degree to which the results of the metric are consistent over time and the level of reproducibility when a similar methodology is adopted (37). Additionally, this study used factor analysis to test the construct validity. Consequently, all the factor loading of the items are above 0.5 (ranging from 0.717 to 0.849), converging into their constructs. Collectively, it suggests that our research framework is significantly valid and consistent.

### Common method bias

According to Podsakoff et al., this study firstly minimized the common method bias during data collection (38). This study designed an appropriate questionnaire length, explicit questions, and anonymous responses (39). Additionally, all the items in the multiple scales were sorted randomly to avoid possible conjecture about the research aim from the respondents. Secondly, Harman's single-factor test as a statistical remedy was used to reduce common method bias (40). The test shows that the largest factor accounts for 42.786% of the overall variance, explaining no single factor to account for the majority of the variance among the measures. Therefore, the common method bias was not a serious problem in this study.

## Results and analysis

### Correlations analysis

Pearson correlation analysis was employed to explore the linear relationships between all the variables. The results are shown in Appendix 2. The correlation matrix indicates that all variables have a stronger positive correlation at the 1% level.

### Regression analysis

Regression analysis is suitable for studying the specific mechanism of action in this research. According to Li, the hierarchical regression analysis is more efficient in estimating the theoretical model with only one mediator (41). Therefore, it was adopted by this research to explore how ethical leadership influences employee wellbeing *via* the mediating role of POS. Table 1 presents the results of the regression analysis.

**Table 1:** Results of regression analysis.

**tblheadVariables**	**POS**		**Employee wellbeing**	
	**Model 1**	**Model 2a**	**Model 2b**	**Model 2c**
**Control variable**				
Gender	−0.091	0.065	0.160	0.091
Age	0.012	0.015	0.008	0.011
Work position	−0.071**	−0.084**	−0.070*	−0.064*
**Independent variable**				
Ethical leadership	0.254***	0.384***		0.312***
**Mediating variable**				
POS			0.427***	0.283**
R^2^	0.194	0.232	0.185	0.271
Adjust R^2^	0.173	0.212	0.163	0.247
F-value	9.050***	11.363***	8.543***	11.560***

*p < 0.05; **p < 0.01; ***p < 0.001; N = 194.

Model 1 in Table 1 shows that ethical leadership is positively related to POS (β = 0.254, *p* < 0.001). Furthermore, it places a positive effect on employee wellbeing shown in Model 2a (β = 0.384, *p* < 0.001). Thus, these results support H1 and H2. Additionally, H3 presents a positive effect of POS on employee wellbeing. Model 2b in Table 1 allows us to accept the hypothesis (β = 0.427, *p* < 0.001).

To test the indirect effect of ethical leadership on wellbeing through POS in H4, this study referred to the four-step procedure by Baron and Kenny (42). For the mediating effect, the first step develops a positive impact of the independent variable (ethical leadership) on the dependent variable (wellbeing). The first step was achieved by the confirmation of H1. The second step was achieved due to the positive effect of the independent variable (ethical leadership) on the mediator variable (POS) in the validated H2. The third step requires the positive impact of the mediator variable (POS) on the dependent variable (wellbeing) to be achieved in the validated H3 (β = 0.427, p <0.001). Finally, we substituted those three types of variables into Model 2c. Followingly, both the independent variable (ethical leadership) and mediating variable (POS) (β = 0.312, *p* < 0.001; β = 0.283, *p* < 0.05) have a positive influence on employee wellbeing. However, the β coefficient of ethical leadership was still remarkable regardless of its slight reduction from 0.384 to 0.312. Furthermore, when the mediating POS is introduced to Model 2c, it results in an adjusted R^2^ of 24.7% that accounts for 24.7% in the variation of employee wellbeing. Despite that, the adjusted R^2^ of Model 2c is fairly increased compared to Model 2a (0.247 > 0.212). The results illustrate that POS partially mediates ethical leadership and employee wellbeing (43–45). Thus, the results strongly support H4.

## Discussions

One finding of this research is the direct effect of ethical leadership on employee wellbeing, which is consistent with prior studies (46–48). This confirms the significant role of ethical leaders in enhancing employee wellbeing to promote organizational performance. The construction industry is infamous for its poor work conditions and incomplete human resources system, consequently, employee wellbeing has long been a critical issue (49, 50). In this case, ethical leaders should concentrate on the needs, feelings, and requirements of employees to enhance their wellbeing. In line with Nawaz Khan et al., project leaders can use ethical-based leadership to motivate their followers by providing safe surroundings and satisfying their needs (12). When their followers understand ethical leadership, they will experience a high level of job satisfaction which predicates improved employee wellbeing (22). Agreeing with previous studies, we propose that ethical leadership is positively related to employee work status, which is an important indicator of employee wellbeing in the construction industry. Therefore, project leaders should be trained to provide constructive feedback, conduct discussions and meetings, and serve employees' needs timely.

Another important finding implies that ethical leadership positively influences POS. Ethical leadership inspires employees' sense of being appreciated and approved by their organizations. When employees receive moral actions and favorable treatment from their leaders, they will fully devote themselves to realize organizational objectives. Consistent with Tan et al., leaders should provide ethical treatments to satisfy employees' emotional and social demands, which would considerably affect POS (25). From transactional perspective, ethical leadership employs explicit ethical standards (such as, rewards and punishment) to guide employees' behaviors (13). For example, they can believe in their employees' abilities, appreciate and reward for their efforts, which would lead to a higher level of POS.

This study also uncovers that POS partially mediates the influence of ethical leadership on employee wellbeing in the construction industry. Expressly, ethical leadership indirectly improves employee wellbeing through POS. Previous studies demonstrated that leaders exert authority or control over their work or project schedules to motivate employees in the workplace (22, 51, 52). Contrarily, we argue that ethical leadership is process-oriented rather than outcome-oriented. Accordingly, ethical leaders should provide organizational support by exhibiting care, fairness, trust, and integrity in their daily project management. Reciprocally, employees will view the leaders' ethical treatments as preferable work experiences, which can enhance POS (33). Based on the COR theory, POS is a socioemotional organizational resource, such as approval, consideration, and respect. Employees will work more diligently when they perceive that their needs are well satisfied and efforts are fairly recognized. Similar with Wen et al., POS, as a critical organizational resource, can help employees develop positive emotions and feelings based on organizational approval of their abilities, support, and understanding of their leaders and coworkers (24). It indicates that the leadership of ethical leaders needs to be reprocessed. Explicitly, ethical leaders' fairness, integrity, and caring should be converted into sufficient organizational resources for employees to increase their wellbeing. This is because employees may have poor fairness due to incomplete performance evaluation in the dynamic work environment (10). They usually desire more organizational recognition for their efforts. Our findings are consistent with Kalshoven and Boon who considered that ethical leadership helps employees gain more organizational resources, which can lead to a higher level of employee wellbeing (44). Therefore, ethical leadership has a remarkable influence on employee wellbeing through POS.

## Conclusions

Although prior research has linked leadership to employee behavioral outcomes, this research highlights the relationship between ethical leadership and employee wellbeing. This research may firstly examine how ethical leadership enhances employee wellbeing *via* POS in the construction industry. Through empirical study, this research verifies the valuable expectation of ethical leadership and demonstrates the significance of ethical leadership in enhancing employee wellbeing. This research provides enlightening references for literature in two ways. First, the positive effect of ethical leadership on employee wellbeing demonstrates that leaders' leadership associated with ethics is vital to improve employee wellbeing in stressful and hazardous environments. Second, the mediating effect of perceived organizational support illustrates that project leaders should pay attention to the accumulation of organizational resources (such as organizational approval of employees' abilities and efforts) in daily life to enhance employee wellbeing in the construction industry.

### Implications

Employees offer a competitive advantage and are a valuable resource for organizational performance, which cannot be achieved without employees' full engagement and high wellbeing. In this sense, we explored how ethical leadership enhances employee wellbeing through POS. Our research can provide an extraordinary practical reference for construction industry to improve their performance.

First, one of the findings demonstrates that ethical leadership positively affects employee wellbeing. In the construction industry, employees usually experience a low level of wellbeing due to tight schedules and daily overloaded work. Employees are likely to suffer from such pressures and typically subject to poor mental health and reduced wellbeing. Undoubtedly, ethical practices and values become vital influencing factors of organizational performance and employees' emotional and psychological states in the construction industry. This research argues that ethical leadership can greatly enhance employee wellbeing through leaders' ethical treatments. Thus, we advise that project management practices in the construction industry should provide leadership training to increase leaders' moral awareness and abilities to implement ethical principles and policies.

Second, this research argues that ethical leadership positively influences POS. In the construction industry, the pursuit of profit typically outweighs employee wellbeing (6), project leaders are suggested to focus more on increasing POS by appreciating and recompensing for employees' efforts. Project leaders should consider employees as valuable capitals of organizations and prioritize their wellbeing, especially mental health. Additionally, project leaders can develop useful mechanisms through which employees can obtain sufficient organizational support, such as creating an organizational supporting environment, to ensure that employees feel supported and encouraged.

Third, our research findings also show that POS plays a mediating role between ethical leadership and employee wellbeing. It implies that project leaders enhance employee wellbeing through POS in a moral manner. They can develop legitimate and transparent reward mechanisms to decrease poor fairness perception of employees and compensate for their emotional needs. Based on the COR theory, ethical leadership accumulates organizational resources. Project leaders in the construction industry should provide ethical and supportive climate to accumulate POS, which enhances employee wellbeing. When employees feel supported, recognized for their contributions, they will feel obligated to achieve organizational goals and long-term development. Since employees gradually become specialists in their professional fields, project leaders should change their management approaches from supervision to encouragement to inspire employees' work enthusiasm and abilities (53). To manage effectively, project leaders can use ethical leadership to accumulate organizational resources to enhance employee wellbeing. Furthermore, this study advises project leaders to perform as ethical rather than unrighteous leaders and gradually change their conventional control-based managerial approaches to integrity-based and caring-based ones to improve satisfaction and engagement.

### Limitations and future research

Although this study suggests adopting ethical leadership to enhance employee wellbeing *via* POS, some limitations still exist. First is the research design. This study relies on a questionnaire-based survey for the targeted population from some construction companies in China. This may not generalize issues of the entire population. Thus, future research should test how our findings could apply to other public domains. Besides, our variables were self-reported, so common method biases may unavoidably exist. We have adopted statistical remedies to test the common source bias. Although they show that the common source was not a serious issue in this study, we expect longitudinal research to decrease any possible common method bias directly, such as collecting data in two phases (25). In addition to the limitations in research design, some questions remain to be further explored. The perception of organizational support is the employees' interpretation of cues conveyed from leaders during the social exchange between leaders and their followers (54). Future research can explore how ethical leadership influences employee wellbeing from a sense-making perspective.

## Data availability statement

The original contributions presented in the study are included in the article/Supplementary material, further inquiries can be directed to the corresponding author.

## Author contributions

JC and LZ proposed the idea and written the overall manuscript. YL and SZ collected data and analyzed the data. All authors read and approved the final manuscript.

## Funding

This work was supported by the Humanity and Social Science Youth Foundation of Ministry of Education of China (No. 19YJC630019), National Natural Science Foundation of China (No. 71872126), and Jiangsu Province University Philosophy and Social Science Research Foundation of China (No. 2017SJB0182).

## Conflict of interest

YL was employed by MCC Huatian Engineering & Technology Corporation. The remaining authors declare that the research was conducted in the absence of any commercial or financial relationships that could be construed as a potential conflict of interest.

## Publisher's note

All claims expressed in this article are solely those of the authors and do not necessarily represent those of their affiliated organizations, or those of the publisher, the editors and the reviewers. Any product that may be evaluated in this article, or claim that may be made by its manufacturer, is not guaranteed or endorsed by the publisher.
