# Supplementary material for: Enhancing employee wellbeing by ethical leadership in the construction industry: The role of perceived organizational support
Source: Front Public Health. 2022 Sep 16;10:935557. doi: 10.3389/fpubh.2022.935557 (PMC9525130; doi:10.3389/fpubh.2022.935557)
Supplement: Supplementary file 1 [file Data_Sheet_1.docx]

**Appendix**

Appendix 1. Demographic and profile of respondents

| Variable | Category | Percentage | Number |
| --- | --- | --- | --- |
| Gender | Male | 76.8% | 149 |
|  | Female | 23.2% | 45 |
| Age | Below 25 | 15.9% | 31 |
|  | 26-35 | 41.8% | 81 |
|  | 36-45 | 32.0% | 62 |
|  | Above 45 | 10.3% | 20 |
| Education | Below junior college | 17.0% | 33 |
|  | Undergraduate | 43.3% | 84 |
|  | Postgraduate | 34.5% | 67 |
|  | PhD | 5.2% | 10 |
| Work position | Senior manager | 6.7% | 13 |
|  | Project manager | 19.6% | 38 |
|  | General employee | 52.1% | 101 |
|  | Senior specialist | 21.6% | 42 |
| Project experience | Below 5 | 34.2% | 66 |
|  | 6-10 | 21.9% | 42 |
|  | 11-15 | 25.0% | 49 |
|  | Above 16 | 18.9% | 37 |

Appendix 2. Correlation matrix for study variables

| Variable name | Mean | SD | EL | POS | EWB |
| --- | --- | --- | --- | --- | --- |
| EL | 3.69 | 0.733 | 1 |  |  |
| POS | 4.24 | 0.563 | 0.345** | 1 |  |
| EWB | 3.54 | 0.728 | 0.408** | 0.381** | 1 |

Note: **p<0.01; N=194.

EL=ethical leadership; POS=perceived organizational support; EWB=employee wellbeing.

Appendix 3. Measurements and reliability

| Construct and items | Factor Loading | Cronbach’s α |
| --- | --- | --- |
| Ethical leadership (EL) |  | 0.933 |
| EL1: My leader listens to what employees have to say. | 0.832 |  |
| EL2: My leader disciplines employees who violate ethical standards. | 0.719 |  |
| EL3: My leader conducts his/her personal life in an ethical manner. | 0.803 |  |
| EL4: My leader has the best interests of employees in mind. | 0.821 |  |
| EL5: My leader makes fair and balanced decisions. | 0.824 |  |
| EL6: My leader can be trusted. | 0.820 |  |
| EL7: My leader discusses business ethics or values with employees. | 0.717 |  |
| EL8: My leader sets an example of how to do things the right way in terms of ethics. | 0.808 |  |
| EL9: My leader defines success not just by results but also the way that they are obtained. | 0.771 |  |
| EL10: My leader when making decisions asks “what is the right thing to do?” | 0.801 |  |
| Perceived organization support (POS) |  | 0.850 |
| POS1: The organization strongly considers my goals and values. | 0.842 |  |
| POS2: The organization really cares about my well-being. | 0.839 |  |
| POS3: If given the opportunity, the organization would take advantage of me. (Reverse) | 0.819 |  |
| POS4: The organization shows great concern for me. | 0.823 |  |
| Employee wellbeing (EWB) |  | 0.937 |
| EWB1: How would you rate your quality of life? | 0.804 |  |
| EWB2: How satisfied are you with your health? | 0.807 |  |
| EWB3: How satisfied are you with your sleep? | 0.834 |  |
| EWB4: How satisfied are you with your ability to perform daily activities? | 0.830 |  |
| EWB5: How satisfied are you with your capacity for work? | 0.849 |  |
| EWB6: How satisfied are you with yourself? | 0.830 |  |
| EWB7: To what extent do you feel that physical pain prevents you from doing what you need to do? | 0.806 |  |
| EWB8: How much do you need any medical treatment to function normally in your daily life? | 0.828 |  |
| EWB9: How often do you have negative feelings such as blue mood, despair, anxiety, depression? | 0.757 |  |
